# Effect of blood transfusion on survival after hip fracture surgery

**DOI:** 10.1007/s00590-018-2205-z

**Published:** 2018-05-11

**Authors:** S. J. M. Smeets, J. P. A. M. Verbruggen, M. Poeze

**Affiliations:** Department of Surgery, Maastricht University Medical Center, Maastricht, The Netherlands

**Keywords:** Blood transfusion, Hip fracture surgery, Survival, Morbidity

## Abstract

**Background:**

Our primary goal was to audit the incidence of erythrocyte blood transfusion (EBT) after hip fracture surgery and study the effects on perioperative complications and early and late mortality.

**Methods:**

In a retrospective cohort study all patients 65 years old and above treated operatively for an acute hip fracture were included over a 48-month period with a 2-year follow-up period. Postoperative hemoglobin levels were used to investigate at what threshold EBT was used. The relation between EBT and perioperative complications and survival was analyzed with multivariate regression analysis. A propensity score for predicting the chance of receiving an EBT was calculated and used to differentiate between transfusion being a risk factor for mortality and other related confounding risk factors. Mortality was subdivided as in-hospital, 30-day, 1-year and 2-year mortality.

**Results:**

Of the 388 included patients, 41% received a blood transfusion. The postoperative hemoglobin level was the strongest predictor for EBT. Patients who received EBT had a significant longer hospital stay and more postoperative cardiac complications, even after adjustment for confounders. Multivariate analysis for mortality showed that EBT was a significant risk factor for early as well as late mortality, but after adding the propensity score, EBT was no longer associated with increased mortality.

**Conclusion:**

There was no effect of EBT on mortality after correction with propensity scoring for predictors of EBT. Transfusion in patients treated operatively for hip fracture should be evenly matched with their cardiovascular risk during the perioperative phase.

## Background

Anemia is very prevalent in patients undergoing surgery for an acute hip fracture (39–69%) [1–6]. Anemia occurs as a result of the trauma, the surgery and can be preexisting in the elderly population. Anemia causes hemodynamic stress, an increased cardiac demand and potential tissue hypoxia in the elderly patients with hip fracture [7–10]. Particularly, those patients with a preexisting cardiac disease are at risk [11]. The incidence of troponin elevation after emergency orthopedic operations varies between 22 and 52.9% [12]. In this context anemia might contribute to the high morbidity and mortality rates after hip surgery. Furthermore, anemia is an independent risk factor for patients not being able to walk postoperatively [2].

Perioperative anemia is mostly treated by erythrocyte blood transfusions (EBT) in order to prevent the postoperative morbidity and mortality. However, there are conflicting data concerning the effect of EBT on the morbidity and mortality in patients with acute hip fractures. Engoren et al. [1] found that allogeneic erythrocyte blood transfusions are associated with an increased long-term mortality (> 90 days up to 2 years) after hip fracture surgery. In addition, increased risk of postoperative infections including pneumonia, delirium, short-term mortality, length of hospital stay and systemic inflammatory response syndrome in patients receiving blood transfusion(s) was reported [4, 13–17]. In contrast, other studies suggest that EBT improves short-term functional outcomes [18–21] and prevents delirium in frail elderly [22, 23].

Another study demonstrated that a restrictive transfusion policy leads to increased cardiovascular events and increased mortality rates [24], although a liberal transfusion trigger did not result in increased ambulation scores or decreased rehabilitation potential. Finally, Carson et al. [25] demonstrated that a liberal transfusion policy was not associated with a lower postoperative morbidity and mortality when compared to a more restrictive transfusion policy in a hip fracture population. Whether EBT is an independent risk factor for the development of postoperative morbidity and mortality besides the clinical factors for giving EBT remains to be seen. Vuille-Lessard et al. [26] found that physicians mostly base their decision to transfuse on the hemoglobin concentration and that characteristics of the patients are minimally considered, although high age, male sex and postoperative hemoglobin levels are other identified predictors for EBT after hip surgery [5, 27, 28].

The aim of this study was to audit the incidence and predictors of receiving a blood transfusion after hip fracture surgery and to investigate the use of blood transfusion as an independent factor on morbidity and mortality. The hypothesis tested in this study was that EBT is not an independent risk factor for morbidity and mortality in patients undergoing surgery for hip fracture, but anemia and underlying cardiovascular disorders are determining the outcome.

## Methods

### Study population

This retrospective study was conducted in the Maastricht University Medical Center in the Netherlands, a level 1 trauma center. All patients from age 65 or above treated operatively for hip fracture were included over a 48-month period. Patients with polytrauma, pathological fractures and patients treated with a total hip arthroplasty were excluded. Patients with a malignancy in their history were not excluded. All patients were operated by the department of trauma surgery using a protocolized treatment algorithm regarding internal fixation (and hip replacement). Duration of follow-up was 2 years. Approval for the study was granted by the Ethical Committee of the Maastricht University Medical Center with a waiver for the need of informed consent.

### Study variables and outcome measurements

All medical records were evaluated for the following content: patient characteristics, fracture type, type of fracture fixation, operation time, delay to surgery, cardiovascular risk factors, preoperative laboratory results, postoperative hemoglobin levels, the use and amount of blood transfusion products, causes of perioperative and postoperative blood loss, postoperative complications and death. Death certificates were obtained from the National population register.

The general transfusion protocol for patients with a cardiac history was to transfuse for hemoglobin levels < 10 g/dl, for elderly without a cardiac history at < 8 g/dl. The majority of patients received postoperative transfusion. Only if the preoperative hemoglobin level was already below the transfusion trigger, a transfusion was given preoperative. The decision to transfuse was made by the attending physician.

Our primary goal was to investigate the association of receiving an EBT and morbidity and mortality after hip fracture surgery. Mortality rates were determined as early in-hospital, 30-day mortality, 1-year and 2-year mortality. Death certificates from the National population register were collected to study postoperative mortality after hospital discharge. To analyze the correlation between cardiac morbidity and EBT, the American College of Cardiology (ACC) and the American Heart Association (AHA) guidelines for perioperative cardiac assessment were used to classify patients cardiac risk into low, intermediate and high risks by clinical risk predictors [29]. We hypothesized that patients with increased cardiac risk would receive more EBT.

### Statistical analysis

All analyses were performed with SPSS 23 statistical software (SPSS Inc., Chicago, Illinois, USA). *p* < 0.05 was considered to be statistically significant. One-way ANOVA was used to compare normally distributed and the Mann–Whitney *U* test for non-normally distributed continuous variables with Bonferroni correction for multiple testing. A Pearson’s Chi-square (*χ*^*2*^) test was used to investigate whether distributions of categorical variables differed from one another. A univariate logistic analysis was performed to identify risk factors for EBT. The likelihood of receiving EBT was calculated with the help of propensity scoring, by logistic regression. The propensity score was used to differentiate between transfusion being a risk factor for mortality and the risk factors for predicting transfusion being predictors for mortality itself. The propensity score was based on all univariate variables significantly associated with transfusion: age, postoperative hemoglobin and fracture type.

To identify possible risk factors for mortality, we used a univariate logistic analysis. A Cox regression analysis was used to determine which regression curve summarizes the best. (Goodness of fit for the regression curve is represented by *R*^2^.) One model was composed with all covariates entered at once (model 1) and one with all the covariates including the propensity score (model 2).

## Results

In the study 388 patients were eligible for inclusion and analyzed. Of these patients, complete data could be collected in 97% of patients. Of 9 (2%) patients time to surgery could not be calculated accurately. Of 4 (1%) patients who lived abroad, follow-up after discharge could not be obtained. These data were regarded as missing data, but other available data of these patients were still used in other analysis.

Table 1 shows the patient and operation characteristics. 41% of patients received a blood transfusion. The transfusion group was significantly older in comparison with patients who did not receive blood transfusion (84 vs 81 years). There was no difference in sex distribution or cardiovascular comorbidity. There were significantly more intracapsular fractures in the non-transfused group. Transfusion was associated with a longer hospital stay (12 vs 10 days).

**Table 1:** Patient and operation characteristics

Patient and operation characteristics (*n* = 388)	EBT (*n* = 160) % (*n*)	No EBT (*n* = 228) % (*n*)	p*
Mean age (years)	84 (SD 7)	81 (SD 7)	**0.001**
Male sex	24% (39)	28% (63)	0.5
Intracapsular fractures	46% (73)	69% (158)	**<0.001**
Extracapsular fractures	54% (87)	31% (70)	
General anesthesia	43% (69)	34% (77)	0.06
Spinal anesthesia	57% (91)	66% (151)	
Median operation time (min)	66 (IQR 33)	66 (IQR 36)	0.1
Median delay to surgery (h)	22 (IQR 19)	22 (IQR 19)	0.8
>24 h	43% (68)	38% (87)	0.4
>48 h	8% (13)	7% (17)	0.8
Cardiac history	49% (79)	43% (97)	0.2
Median patient days (*n*)	12 (IQR 13)	10 (IQR 8)	**0.01**
Preoperative hemoglobin (g/dl)	12.4 (IQR 0.9)	13.4 (IQR 1.2)	0.4
Postoperative hemoglobin (g/dl)	7.7 (IQR 0.8)	10.1 (IQR 1.3)	**<0.001**
Median number of transfusions	2.0	–	–

Significant values are in bold

*SD* standard deviation, *IQR* interquartile range

*p value, significant for *p* < 0.05; by univariate analyses between EBT and no EBT

The median number of blood transfusion was 2.0 with a median postoperative hemoglobin level of 7.7 g/dl. The median postoperative hemoglobin level in the non-transfused group was significantly higher (10.0 g/dl, *p* < 0.001). Postoperative hemoglobin levels were strongly associated with use of EBT (both in univariate and in multivariate analyses, *p* < 0.001). Other factors associated with transfusion were high age and intracapsular fractures. Sex, a cardiac history, delay to surgery and operation time were not associated with EBT. Multivariate analysis showed postoperative hemoglobin level, a cardiac history and delay to surgery > 48 h as significant risk factors for in-hospital mortality (*p* < 0.01). Postoperative anemia remained a predictor for 30-day, 1-year and 2-year mortality, both in univariate and in multivariate analyses.

### Cardiac risk

A cardiac history was present in 45.4% of patients. In total 37 patients were predicted to have a high perioperative complication risk, 113 an intermediate risk and 238 a low risk using the ACC/AHA guidelines. Patients with high cardiac risk did not receive more transfusions than patients with intermediate (*p* = 0.08) or low cardiac risk (*p* = 0.06) although there seems to be a trend. High-risk patients had significant more cardiovascular complications after surgery and risk of mortality.

### EBT and morbidity

Patients who received EBT were at risk of postoperative complications (see Table 2): delirium (RR 1.5 95% CI 1.2–2.0; *p* = 0.002), gastrointestinal bleeding (RR 14 95% CI 1.9–110.0; *p* = 0.001), respiratory complications (RR 2.4 95% CI 1.3–4.4; *p* = 0.004) and cardiovascular complications (RR 3.8, 95% CI 1.9–8.0; *p* < 0.001). Even after correction for sex, age, fracture type, delay to surgery, cardiac history and postoperative hemoglobin levels did patients with EBT experience more cardiovascular complications. There was no dose–effect found between number of transfused units and morbidity or mortality when divided into three groups: 1–2, 3–4 or > 4 transfused units.

**Table 2:** Complications and mortality

Complications	EBT % (*n*)	No EBT % (*n*)	p*
Cardiovascular^b^	15% (24)	4% (9)	**<0.001**
Stroke	3% (4)	1% (2)	0.2
Rhythm disorders	5% (8)	1% (2)	**0.01**
AMI	6% (9)	1% (3)	**0.02**
Cardiac failure	3% (4)	3% (6)	0.9
Respiratory^a^	16% (25)	7% (15)	**0.004**
Pneumonia	11% (18)	6% (14)	0.07
Pulmonary embolism	3% (4)	(0)	**0.02**
Respiratory failure	6% (10)	0.4% (1)	**0.001**
Delirium	46% (73)	37% (84)	**0.002**
Wound infection	9% (14)	5% (11)	0.1
Urinary tract infection	16% (25)	19% (44)	0.4
Pressure sores	18% (29)	10% (22)	0.02
Gastrointestinal tract bleeding	6% (10)	0.4% (1)	**0.001**
DVT	3% (5)	1% (3)	0.2
In-hospital mortality	8% (12)	3% (6)	**0.03**
30-Day mortality	14% (23)	4% (10/224)	**0.001**
1-Year mortality	37% (59)	22% (50/224)	**0.002**
2-Year mortality	47% (75)	33% (74/224)	**0.006**

Significant values are in bold

^a^Respiratory complications consist of pneumonia, pulmonary embolism and respiratory failure together

^b^Cardiovascular complications consist of stroke, rhythm disorders, acute myocardial infarction and cardiac failure. *DVT* deep venous thrombosis

*p value, significant for *p* < 0.05; by univariate analyses between EBT and no EBT

### EBT and mortality

A univariate analysis for in-hospital, 30-day, 1-year and 2-year mortality showed that sex, age, a delay to surgery of > 48 h, a cardiac history and the use of blood transfusion were significant risk factors. These variables were entered in a Cox regression model (model 1) with 24 months of follow-up. Sex, age, delay to surgery > 48 h and EBT were significant risk factors for mortality (see Table 3). After adding the propensity score as a variable in the model (model 2), EBT was no longer significant. 38% (149/388) of patients were dead at 2-year follow-up. 50% (75/149) of the deceased patients received EBT compared to 36% (85/239) who survived (*p* = 0.001). Sex and age were found the only predictors for mortality within 2 years.

**Table 3:** Cox regression model for mortality

Mortality	Covariates	Cox regression model 1^a^	Exp (B) (95% CI)	Cox regression model 2^b^	Exp (B) (95% CI)
		*p*		*p*	
In-hospital	Age	0.6	1.0 (1.0–1.1)	0.8	1.0 (0.9–1.0)
	Sex	0.2	1.8 (0.7–4.5)	0.1	2.2 (0.9–5.7)
	Delay > 48 h	0.08	0.4 (0.1–1.1)	**<0.05**	0.3 (0.1–1.0)
	Cardiac history	**0.02**	0.3 (0.1–0.8)	**0.03**	0.3 (0.1–0.9)
	EBT	**0.04**	0.4 (0.1–0.9)	0.9	1.0 (0.3–3.6
	Propensity score	–	–	0.02	9.5 (1.5–62)
30-Day	Age	0.1	1.0 (10–1.1)	0.2	1.0 (1.0–1.1)
	Sex	0.6	1.2 (0.6–2.6)	0.6	1.2 (0.6–2.7)
	Delay > 48 h	**0.04**	0.4 (0.2–1.0)	**0.02**	0.3 (0.1–0.8)
	Cardiac history	0.1	0.6 (0.3–1.1)	0.1	0.6 (0.3–1.2)
	EBT	**0.004**	0.3 (0.2–0.7)	0.7	0.8 (0.3–2.2)
	Propensity score	–	–	0.02	5.2 (1.3–22)
1-Year	Age	**<0.001**	1.0 (1.0–1.1)	**0.001**	1.0 (1.0–1.1)
	Sex	**0.001**	1.9 (1.3–2.8)	**<0.001**	2.1 (1.4–3.2
	Delay > 48 h	0.1	0.6 (0.3–1.1)	**0.04**	0.6 (0.3–1.0)
	Cardiac history	0.1	0.7 (0.5–1.1)	0.1	0.7 (0.5–1.1)
	EBT	**0.004**	0.6 (0.4–0.8)	0.7	0.9 (0.5–1.6)
	Propensity score	–	–	0.04	2.3 (1.0–5.1)
2-Year	Age	**<0.001**	1.0 (1.0–1.1)	**<0.001**	1.0 (1.0–1.1)
	Sex	**<0.001**	2.1 (1.5–3.0)	**<0.001**	2.3 (1.6–3.3)
	Delay > 48 h	0.4	0.8 (0.4–1.4)	0.2	0.7 (0.4–1.3)
	Cardiac history	**0.03**	0.7 (0.5–1.0)	0.06	0.7 (0.5–1.0)
	EBT	**0.01**	0.7 (0.5–0.9)	0.3	0.8 (0.4–1.3)
	Propensity score	–	–	0.4	1.4 (0.7–2.8)

Significant values are in bold

^a^Model 1: all significant covariates for mortality entered in Cox regression analysis

^b^Model 2: all covariates including the propensity score. *Exp(B)* exponentiation of the B coefficient, an odds ratio. 95% CI = 95% confidence interval. *p* value, significant for *p* < 0.05

## Discussion

The hypothesis tested in this study was that the use of erythrocyte blood transfusion (EBT) in patients treated operatively for hip fracture is not a risk factor for mortality but depends primarily on the effects of anemia and underlying cardiovascular disorders. We found that 41% of patients with hip fracture received EBT. Our hospital practiced a liberal transfusion policy to transfuse elderly patients with a cardiac history (aimed at > 10 g/dL). Despite this, no differences existed in the frequency of EBT administered when patients were classified into low, intermediate and high cardiac risk. This study demonstrated that EBT is frequently used during the treatment of acute anemia after hip fracture surgery. The association of EBT on postoperative mortality was no longer present after correction with the propensity score for receiving a transfusion. However, EBT remained an independent risk of perioperative cardiac morbidity.

This is in contradiction with the results of Engoren et al. [1] who used propensity matching and found a significantly increased relative risk of 3.8 (95% CI 1.2–11.6) for EBT on mortality. In our study we used the propensity score for receiving EBT as a variable in our Cox model in attempt to correct for the chance of receiving EBT instead of adding to many individual variables in the model. Another study showed that risk of mortality after EBT was significantly higher when the preoperative hemoglobin level was 10 g/dL or less in patients with preexistent cardiovascular disease [30]. Patients with low hemoglobin levels are more likely to suffer from cardiac complications in case of preexistent cardiac comorbidity [11].

A review from Potter et al. [31] on preoperative anemia and EBT in hip fracture patients showed that anemia on hospital admission was associated with increased mortality, relative risk 1.64 (95% CI 1.47–1.82), *p* < 0.0001 in > 13.000 patients. There was no association between postoperative transfusion and mortality after adjusting for covariates. These findings suggest that anemia seems to play a more important role than EBT itself. This is exactly why we corrected our data with the use of the propensity score for the chance of receiving EBT.

In our study, EBT remained an independent risk of perioperative cardiac morbidity after correction for confounders. In a large RCT from Carson et al., the rates of in-hospital acute coronary syndrome or death were 4.3 and 5.2%, in the liberal and restrictive transfusion group, respectively (absolute risk difference, −0.9%; 99% CI −3.3 to 1.6) [25]. In Potters review transfusion at 80 vs 100 g/dl increased acute myocardial infarction, with a relative risk of 1.67 (95% CI 1.01–2.77), although with *p* = 0.05 and a 97.5% weight of Carson’s study [25]. No strong conclusions can be drawn.

The TRIFE trial showed comparable findings between liberal and restrictive blood transfusion protocols after hip fracture surgery, but subgroup analyses for frail elderly in nursing homes showed that this group could benefit from a more liberal transfusion [32]. Post hoc analyses showed furthermore a reduced risk of postoperative delirium for the liberal transfusion group. Development of delirium on day 10 postoperatively after hip fracture surgery increased the risk of 90-day death, hazard ratio 3.14 (95% CI 1.72–5.78, *p* < 0.001) [33].

A number of remarks must be made when interpreting the results of this study. Selection bias cannot be excluded because of the retrospective study design. It is possible that other contributing risk factors were not identified in this study and therefore not taken into account in the analyses. A limitation of propensity matching is that a variable that affects treatment assignment but not outcome is analyzed the same as a variable that affects treatment assignment and has a strong relationship to outcome as well [34]. This suggests that risk factors for transfusion are predictors for mortality itself. And indeed, age and postoperative hemoglobin levels were associated with early as well as late mortality. We found no association between mortality and fracture type. Another study found no effect of EBT on mortality after hip fracture surgery when corrected for age, male sex, residential status, preoperative hemoglobin level, mobility score and American Society of Anesthesiologists score [18].

The mechanisms by which blood transfusion might worsen outcomes are unknown. There is evidence that the storage time of erythrocytes is associated with increased mortality [35–39]. Koch et al. [37] showed that cardiac surgical patients receiving erythrocytes that had been stored for more than 2 weeks have a higher risk of in-hospital mortality and postoperative complications. Storage leads to numerous effects: decreased cellular deformability, increased adhesion to the vascular endothelium [40], impaired microvascular flow [30, 41] and abnormal shape [42] which leads to limited oxygen delivery.

Another pathway is transfusion-induced immunomodulation (TRIM) and focuses on the release of cytokines, allogenic leukocytes and their immune activation, resulting in transfusion-related lung injury or immune suppression resulting in susceptibility to infectious complications [41, 43–45]. The fact that the association between EBT and mortality is not present on the long-term mortality may indicate that such processes are contributing.

Another extensively studied phenomenon is transfusion-related acute lung injury (TRALI). The diagnosis must satisfy the criteria for acute lung injury (ALI) following ≤ 6 h after transfusion, including (1) acute onset, (2) hypoxemic lung disease with (3) bilateral infiltrates on frontal chest radiograph and (4) no evidence of left atrial hypertension [46].

Anemia contributes to and is associated with reduced physiological/homeostatic reserve, reduced function and reduced mobility [2, 47], mechanisms through which complications and mortality may be increased beyond 1 year postoperative. The ‘frailty syndrome,’ consisting of sarcopenia, anorexia and declining mobility, is associated with dysregulation of pro-inflammatory pathways, increasing inflammatory markers such as IL-6, bone marrow suppression and chronic anemia [48].

EBT was not associated with early or late mortality after hip fracture surgery. Postoperative anemia, a cardiac history and delay to surgery of > 48 h were predictors for in-hospital mortality. Transfusion in patients treated operatively for hip fracture should be evenly matched with their cardiovascular risk during the perioperative phase. Further research should identify subgroups that may benefit from a more liberal transfusion trigger depending on their cardiac risk and frailty characteristics or a more restrictive transfusion trigger without risk of worse outcome.

## References

[1] Engoren M, Mitchell E, Perring P, Sferra J (2008). The effect of erythrocyte blood transfusions on survival after surgery for hip fracture. J Trauma.

[2] Foss NB, Kristensen MT, Kehlet H (2008). Anaemia impedes functional mobility after hip fracture surgery. Age Ageing.

[3] Garcia-Erce JA, Cuenca J, Haman-Alcober S, Martinez AA, Herrera A, Munoz M (2009). Efficacy of preoperative recombinant human erythropoietin administration for reducing transfusion requirements in patients undergoing surgery for hip fracture repair. An observational cohort study. Vox Sang.

[4] Spahn DR (2010). Anemia and patient blood management in hip and knee surgery: a systematic review of the literature. Anesthesiology.

[5] Verlicchi F, Desalvo F, Zanotti G, Morotti L, Tomasini I (2011). Red cell transfusion in orthopaedic surgery: a benchmark study performed combining data from different data sources. Blood Transfus.

[6] Carson JL, Terrin ML, Barton FB, Aaron R, Greenburg AG, Heck DA, Magaziner J, Merlino FE, Bunce G, McClelland B, Duff A, Noveck H (1998). A pilot randomized trial comparing symptomatic vs. hemoglobin-level-driven red blood cell transfusions following hip fracture. Transfusion.

[7] Foss NB, Kehlet H (2006). Hidden blood loss after surgery for hip fracture. J Bone Joint Surg Br.

[8] Marval PD, Hardman JG (2004). Perioperative blood loss and transfusion requirements in patients with fractured neck of femur. Eur J Anaesthesiol.

[9] Carson JL, Terrin ML, Jay M (2003). Anemia and postoperative rehabilitation. Can J Anaesth.

[10] Kehlet H, Dahl JB (2003). Anaesthesia, surgery, and challenges in postoperative recovery. Lancet.

[11] Carson JL, Duff A, Poses RM, Berlin JA, Spence RK, Trout R, Noveck H, Strom BL (1996). Effect of anaemia and cardiovascular disease on surgical mortality and morbidity. Lancet.

[12] Chong CP, van Gaal WJ, Savige J, Lim WK (2011). Cardiac injury and troponin testing after orthopaedic surgery. Injury.

[13] Pedersen AB, Mehnert F, Overgaard S, Johnsen SP (2009). Allogeneic blood transfusion and prognosis following total hip replacement: a population-based follow up study. BMC Musculoskelet Disord.

[14] Bierbaum BE, Callaghan JJ, Galante JO, Rubash HE, Tooms RE, Welch RB (1999). An analysis of blood management in patients having a total hip or knee arthroplasty. J Bone Joint Surg Am.

[15] Carson JL, Altman DG, Duff A, Noveck H, Weinstein MP, Sonnenberg FA, Hudson JI, Provenzano G (1999). Risk of bacterial infection associated with allogeneic blood transfusion among patients undergoing hip fracture repair. Transfusion.

[16] Dunne JR, Malone DL, Tracy JK, Napolitano LM (2004). Allogenic blood transfusion in the first 24 hours after trauma is associated with increased systemic inflammatory response syndrome (SIRS) and death. Surg Infect (Larchmt).

[17] Guo Y, Jia P, Zhang J, Wang X, Jiang H, Jiang W (2016). Prevalence and risk factors of postoperative delirium in elderly hip fracture patients. J Int Med Res.

[18] Johnston P, Wynn-Jones H, Chakravarty D, Boyle A, Parker MJ (2006). Is perioperative blood transfusion a risk factor for mortality or infection after hip fracture?. J Orthop Trauma.

[19] Halm EA, Wang JJ, Boockvar K, Penrod J, Silberzweig SB, Magaziner J, Koval KJ, Siu AL (2003). Effects of blood transfusion on clinical and functional outcomes in patients with hip fracture. Transfusion.

[20] Carson JL, Duff A, Berlin JA, Lawrence VA, Poses RM, Huber EC, O’Hara DA, Noveck H, Strom BL (1998). Perioperative blood transfusion and postoperative mortality. JAMA.

[21] Gregersen M (2016). Postoperative red blood cell transfusion strategy in frail anemic elderly with hip fracture. A randomized controlled trial. Dan Med J.

[22] Blandfort S, Gregersen M, Borris LC, Damsgaard EM (2016). Blood transfusion strategy and risk of postoperative delirium in nursing homes residents with hip fracture.

[23] van der Zanden V, Beishuizen SJ, Scholtens RM, de Jonghe A, de Rooij SE, van Munster BC (2016). The effects of blood transfusion on delirium incidence. J Am Med Dir Assoc.

[24] Foss NB, Kristensen MT, Jensen PS, Palm H, Krasheninnikoff M, Kehlet H (2009). The effects of liberal versus restrictive transfusion thresholds on ambulation after hip fracture surgery. Transfusion.

[25] Carson JL, Terrin ML, Noveck H, Sanders DW, Chaitman BR, Rhoads GG, Nemo G, Dragert K, Beaupre L, Hildebrand K, Macaulay W, Lewis C, Cook DR, Dobbin G, Zakriya KJ, Apple FS, Horney RA, Magaziner J, F Investigators (2011). Liberal or restrictive transfusion in high-risk patients after hip surgery. N Engl J Med.

[26] Vuille-Lessard E, Boudreault D, Girard F, Ruel M, Chagnon M, Hardy JF (2010). Red blood cell transfusion practice in elective orthopedic surgery: a multicenter cohort study. Transfusion.

[27] Gombotz H, Rehak PH, Shander A, Hofmann A (2007). Blood use in elective surgery: the Austrian benchmark study. Transfusion.

[28] Goodnough LT, Vizmeg K, Sobecks R, Schwarz A, Soegiarso W (1992). Prevalence and classification of anemia in elective orthopedic surgery patients: implications for blood conservation programs. Vox Sang.

[29] Fleisher LA, Beckman JA, Brown KA, Calkins H, Chaikof E, Fleischmann KE, Freeman WK, Froehlich JB, Kasper EK, Kersten JR, Riegel B, Robb JF, Smith SC, Jacobs AK, Adams CD, Anderson JL, Antman EM, Buller CE, Creager MA, Ettinger SM, Faxon DP, Fuster V, Halperin JL, Hiratzka LF, Hunt SA, Lytle BW, Nishimura R, Ornato JP, Page RL, Tarkington LG, Yancy CW (2008). ACC/AHA 2007 guidelines on perioperative cardiovascular evaluation and care for noncardiac surgery: executive summary: a report of the American College of Cardiology/American Heart Association Task Force on Practice Guidelines (Writing Committee to Revise the 2002 Guidelines on Perioperative Cardiovascular Evaluation for Noncardiac Surgery). Anesth Analg.

[30] Klein HG, Spahn DR, Carson JL (2007). Red blood cell transfusion in clinical practice. Lancet.

[31] Potter LJ, Doleman B, Moppett IK (2015). A systematic review of pre-operative anaemia and blood transfusion in patients with fractured hips. Anaesthesia.

[32] Gregersen M, Borris LC, Damsgaard EM (2015). Postoperative blood transfusion strategy in frail, anemic elderly patients with hip fracture: the TRIFE randomized controlled trial. Acta Orthop.

[33] Blandfort S, Gregersen M, Borris LC, Damsgaard EM (2017). Blood transfusion strategy and risk of postoperative delirium in nursing homes residents with hip fracture. A post hoc analysis based on the TRIFE randomized controlled trial. Aging Clin Exp Res.

[34] Rubin DB (1997). Estimating causal effects from large data sets using propensity scores. Ann Intern Med.

[35] Basran S, Frumento RJ, Cohen A, Lee S, Du Y, Nishanian E, Kaplan HS, Stafford-Smith M, Bennett-Guerrero E (2006). The association between duration of storage of transfused red blood cells and morbidity and mortality after reoperative cardiac surgery. Anesth Analg.

[36] Engoren MC, Habib RH, Zacharias A, Schwann TA, Riordan CJ, Durham SJ (2002). Effect of blood transfusion on long-term survival after cardiac operation. Ann Thorac Surg.

[37] Koch CG, Li L, Sessler DI, Figueroa P, Hoeltge GA, Mihaljevic T, Blackstone EH (2008). Duration of red-cell storage and complications after cardiac surgery. N Engl J Med.

[38] Bennett-Guerrero E, Stafford-Smith M, Waweru PM, Bredehoeft SJ, Campbell ML, Haley NR, Phillips-Bute B, Newman MF, Bandarenko N (2009). A prospective, double-blind, randomized clinical feasibility trial of controlling the storage age of red blood cells for transfusion in cardiac surgical patients. Transfusion.

[39] Eikelboom JW, Cook RJ, Liu Y, Heddle NM (2010). Duration of red cell storage before transfusion and in-hospital mortality. Am Heart J.

[40] Tinmouth A, Fergusson D, Yee IC, Hebert PC (2006). Clinical consequences of red cell storage in the critically ill. Transfusion.

[41] Raghavan M, Marik PE (2005). Anemia, allogenic blood transfusion, and immunomodulation in the critically ill. Chest.

[42] Berezina TL, Zaets SB, Morgan C, Spillert CR, Kamiyama M, Spolarics Z, Deitch EA, Machiedo GW (2002). Influence of storage on red blood cell rheological properties. J Surg Res.

[43] Blajchman MA (2005). Transfusion immunomodulation or TRIM: what does it mean clinically?. Hematology.

[44] Zallen G, Moore EE, Ciesla DJ, Brown M, Biffl WL, Silliman CC (2000). Stored red blood cells selectively activate human neutrophils to release IL-8 and secretory PLA2. Shock.

[45] Vamvakas EC, Blajchman MA (2001). Deleterious clinical effects of transfusion-associated immunomodulation: fact or fiction?. Blood.

[46] Bernard GR, Artigas A, Brigham KL, Carlet J, Falke K, Hudson L, Lamy M, Legall JR, Morris A, Spragg R (1994). The American-European consensus conference on ARDS. Definitions, mechanisms, relevant outcomes, and clinical trial coordination. Am J Respir Crit Care Med.

[47] Lawrence VA, Silverstein JH, Cornell JE, Pederson T, Noveck H, Carson JL (2003). Higher Hb level is associated with better early functional recovery after hip fracture repair. Transfusion.

[48] Roy CN (2011). Anemia in frailty. Clin Geriatr Med.

